# Nanomodified Peek Dental Implants: Bioactive Composites and Surface Modification—A Review

**DOI:** 10.1155/2015/381759

**Published:** 2015-10-01

**Authors:** Shariq Najeeb, Zohaib Khurshid, Jukka Pekka Matinlinna, Fahad Siddiqui, Mohammad Zakaria Nassani, Kusai Baroudi

**Affiliations:** ^1^Restorative Dental Sciences, Al-Farabi Colleges, King Abdullah Road, P.O. Box 85184, Riyadh 11891, Saudi Arabia; ^2^School of Metallurgy and Materials, University of Birmingham, Edgbaston, Birmingham B15 2TT, UK; ^3^Dental Materials Science, Faculty of Dentistry, The University of Hong Kong, 4/F, The Prince Philip Dental Hospital, 34 Hospital Road, Sai Ying Pun, Hong Kong; ^4^Division of Oral Health & Society, 2001 McGill College, Suite 500, Montreal, QC, Canada H3A 1G1; ^5^Preventive Dental Sciences, Al-Farabi Colleges, King Abdullah Road, P.O. Box 85184, Riyadh 11891, Saudi Arabia

## Abstract

*Purpose*. The aim of this review is to summarize and evaluate the relevant literature regarding the different ways how polyetheretherketone (PEEK) can be modified to overcome its limited bioactivity, and thereby making it suitable as a dental implant material. *Study Selection*. An electronic literature search was conducted via the PubMed and Google Scholar databases using the keywords “PEEK dental implants,” “nano,” “osseointegration,” “surface treatment,” and “modification.” A total of 16 *in vivo* and *in vitro* studies were found suitable to be included in this review. *Results*. There are many viable methods to increase the bioactivity of PEEK. Most methods focus on increasing the surface roughness, increasing the hydrophilicity and coating osseoconductive materials. *Conclusion*. There are many ways in which PEEK can be modified at a nanometer level to overcome its limited bioactivity. Melt-blending with bioactive nanoparticles can be used to produce bioactive nanocomposites, while spin-coating, gas plasma etching, electron beam, and plasma-ion immersion implantation can be used to modify the surface of PEEK implants in order to make them more bioactive. However, more animal studies are needed before these implants can be deemed suitable to be used as dental implants.

## 1. Introduction

A dental subgingival implant is a fixture, surgically placed into the alveolar bone, which functions as an artificial root that can stabilize and support a fixed or removable prosthesis [1, 2]. In general, after implantation of a biomaterial, two possible tissue responses can take place. If a fibrous tissue forms between the implant and the bone, the implant fails. However, if a direct intimate bone-implant contact forms, the implant is said to be osseointegrated (a.k.a. osteointegrated) into the alveolar bone [3]. Osseointegration depends on a number of factors. As described by Brånemark [4] and Albrektsson et al. [3], implant material, surgical technique, and healing period are the main factors which govern the success of dental implants. The implant material, usually titanium and its alloys [5], zirconia [6], or, as a potential future material, fiber reinforced composite (FRC) [7] should be biocompatible [8] and should possess suitable surface properties that induce bone formation around the implant. The implant material should have a suitable design [9], high hydrophilicity [10], and an appropriate surface roughness [11]. Coating the implant surface with osteoconductive coatings such as calcium phosphate [12] has been shown to increase the rate of osseointegration of dental implants [11]. Over the last several decades, commercially pure grade 2 or 4 titanium and its alloys have been the material of choice for endosseous implants [13]. However, titanium has been shown to exhibit a variety of problems. Because of the high modulus of elasticity of the titanium alloys, dental implants made from the material can cause stress-shielding [14] which may lead to periodontal bone loss [15]. Moreover, studies have documented very rare cases of patients developing hypersensitivity to titanium dental implants [16, 17]. Wear debris and ion leakage [18] can also be of concern with titanium dental implants. Aesthetics can be compromised if the dental implant is visible through a thin biotype gingiva because titanium is a dark material.

**Figure 1:**
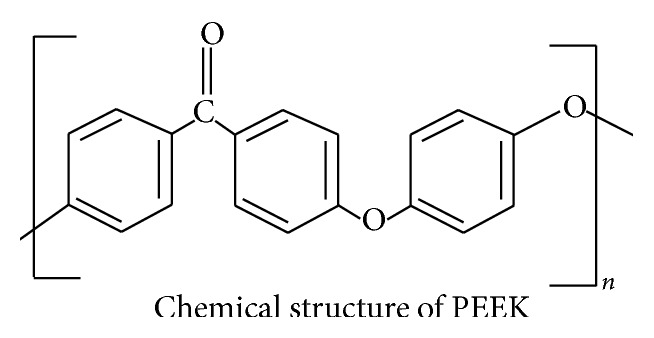
The chemical structural formula of polyetheretherketone (PEEK). PEEK is a semicrystalline thermoplastic and it is synthesized via step-growth polymerization by the dialkylation of* bis*-phenolate salts.

Polyetheretherketone (PEEK) is an organic synthetic polymeric tooth coloured material which has the potential to serve as an aesthetic dental implant material [19]. The structure of PEEK is given in Figure 1. It has excellent chemical resistance and biomechanical properties. In its pure form, Young's modulus of PEEK is around 3.6 GPa. Meanwhile, Young's modulus of carbon-reinforced PEEK (CFR-PEEK) is around 18 GPa [7] which is close to that of cortical bone [20, 21]. Hence, it has been suggested that PEEK could exhibit lesser stress-shielding when compared to titanium [22]. However, PEEK has been shown to stimulate less osteoblast differentiation when compared to titanium [23]. This said, PEEK is a bioinert material and it does not possess any inherent osseoconductive properties [24]. PEEK can be coated and blended with bioactive particles to increase the osseoconductive properties and surface roughness. However, high temperatures involved in plasma-spraying can deteriorate PEEK. Furthermore, thick calcium phosphate coatings on PEEK can delaminate because of their limited bond strength when compared to coated titanium implants [25, 26]. Additionally, combining PEEK with particles in the size range of micrometers leads to mechanical properties falling inferior to those of pure PEEK or CFR-PEEK [27]. Therefore, more recently, a significant amount of research has been conducted to modify PEEK by coating or blending it with nanosized particles and producing nanolevel surface topography. The aim of this review is to highlight recent advancements towards producing bioactive nanocomposites and nanolevel surface modifications to ascertain the feasibility of nanomodified PEEK to be used as dental implant material.

## 2. Bioactive PEEK Nanocomposites

Bioactive particles can be incorporated into PEEK to produce bioactive implants [27]. Hydroxyapatite is a bioceramic with chemistry similar to bone and it is shown to induce bone formation around implants [11]. Hydroxyapatite particles (HAp) of the micrometer size range have been melt-blended with PEEK producing PEEK-HAp composites but these could be very difficult to be used as dental implants because of the poor mechanical properties produced due to the insufficient interfacial bonding between PEEK and hydroxyapatite particles [27, 28].

**Figure 2:**
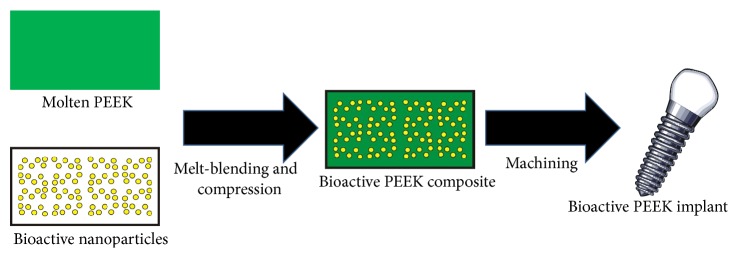
A schematic diagram of the process of melt-blending to produce bioactive PEEK composites. First, the PEEK powder and nanofillers are codispersed in a suitable solvent. The solvent is then removed and the mixture is placed in suitable moulds and heated to a temperature above the melting point PEEK under high pressure in a mould (so-called compression moulding). Upon cooling, the resultant material is composite of PEEK and the fillers. The solid composite is then machined to provide shape or surface characteristics suitable for a dental implant.

Melt-blending PEEK with nanoparticles can be achieved to produce bioactive composite PEEK composite implants and at the same time enhance their mechanical properties [29]. The schematic diagram of the melt-blending process is shown in Figure 2. Melt-blending of PEEK with bioactive nanofillers has been described by Wan et al. [29] and Wu et al. [30]. First, the PEEK powder and nanofillers are codispersed in a suitable solvent to form a uniform suspension. The solvent is then removed by drying in an oven and the powder mixture is placed in suitable moulds in the shape of the implants. The powder mixture and the moulds are preheated to a temperature of about 150°C at 35 MPa pressure. The temperature is then increased to 350°C–400°C at 15 MPa. When the melting point of PEEK is reached, the polymer melts but the bioactive filler particles remain solid. The temperature is maintained for 10 min after which the composite implants are air-cooled to 150°C. Upon cooling, the resultant material is a composite of solid PEEK matrix and the nanofillers dispersed within it (Figure 2).

As shown in Table 1, it can be observed that incorporating nanosized particles to PEEK can produce PEEK composites with enhanced mechanical properties and bioactivity. Wu et al. have suggested that incorporating nanosized TiO_2_ particles to PEEK can increase osseointegration [30]. Three-dimensional computerized tomography has shown that a higher amount of bone forms around PEEK/nano-TiO_2_ cylindrical implants and they have improved mechanical properties when compared to pure PEEK because of an increased number of nanofiller particles [30]. The effect of free TiO_2_ particles on cellular activity has been debated in the literature. Some studies suggest that they can stimulate an inflammatory or carcinogenic response in cells [31] and damage nerve tissue [32]. On the other hand, some studies have suggested that, when used as coatings or solid cores, TiO_2_ can increase the rate of cellular proliferation and differentiation [33–35]. However, to date, no studies have investigated the possible release of TiO_2_ particles from PEEK/nano-TiO_2_ composites after undergoing mechanical loading.

Fluorohydroxyapatite (HAF) has been shown to induce higher bone cell proliferation than conventional hydroxyapatite and it possesses antibacterial properties due to the presence of fluoride ions (F^−^) [36–40]. Wang et al. have shown that it can be possible to produce PEEK/nano-HAF implants using the process of melt-blending [29]. These implants possess antimicrobial properties against* Streptococcus mutans*, one of the main causative agents of periodontitis, and can exhibit Young's modulus almost 3 times of that of pure PEEK [29]. This increased modulus is still near to that of bone so PEEK/nano-HAF implants could still produce less stress-shielding than titanium implants. However, no studies have been attempted to investigate this.

Even with the incorporation of bioactive nanoparticles, the water-contact angle of PEEK nanocomposites does not decrease significantly when compared to pure PEEK [29, 30]. Although gas plasma treatment of PEEK/nano-HAp composite implants does reduce the surface contact angle to as low as 10°, they have not been tested* in vitro* and the effects of plasma gas treatment have been seen to be temporary [41]. As an increased contact angle is an indication of a more hydrophobic implant surface [11], it is still uncertain whether a high contact angle can undermine the long-term biocompatibility of these implants and more research is warranted to investigate this concern.

## 3. Surface Modifications of PEEK Implants

Different types of surface modifications geared towards making PEEK more bioactive are summarized in Table 2. In contrast to production of nanocomposites of PEEK, surface modification aims to alter the surface of PEEK with little or no effect on the core. To date, four processes have been used to nanomodify the surface of PEEK implants: spin-coating [42–44], gas plasma etching [45–52], electron beam deposition [53–57], and plasma-ion immersion implantation (PIII) [58–62].

### 3.1. Spin-Coating with Nanohydroxyapatite

Due to the drawbacks of thick hydroxyapatite coatings, research has been conducted and directed to coat implants with thinner coatings [11]. Spin-coating involves the deposition of a thin layer of nano-HA, precipitated in surfactants, organic solvents, and aqueous solution of Ca(NO_3_)_2_ and H_3_PO_4_, on the implants. During the deposition, the implants are spun at high speeds and are then heat-treated to form the coating [42]. The first study evaluating spin-coated PEEK implants by Barkarmo et al. [42] showed that the mean removal torque of spin-coated implanted discs was not significantly greater than that of uncoated implants and during the study, several implants failed. However, subsequent studies by Barkarmo et al. [43] and Johansson et al. [44] found higher removal torques compared to uncoated PEEK when the implant design had been modified by adding a threaded, cylindrical design. The findings suggested that an appropriate implant design is a very important factor as well as a suitable bioactive coating for successful PEEK dental subgingival implants. Nevertheless, there have been no studies conducted testing the bond strength of the nanohydroxyapatite coatings and all the current studies on spin-coated nanohydroxyapatite implants have not found any significant differences in the bone-implant contact of the modified and unmodified PEEK.

### 3.2. Gas Plasma Nanoetching

Nanoetching of PEEK implants can be achieved by exposing them to low power plasma gases like water vapour [45], oxygen/argon, and ammonia [46, 47]. It has been suggested that plasma treatment of PEEK introduces various functional groups on its surface which makes its surface more hydrophilic [48]. The main advantage of using plasma treatment is the ability to produce nanolevel roughness on the implant surface and the extremely low water-contact angle on PEEK surface [46]. Indeed,* in vitro* testing of plasma-etched PEEK implants has been shown to accelerate human mesenchymal cell proliferation [46]. This has been thought to occur because of the increased hydrophilicity [49] and protein adsorption due to nanoroughness [50]. Because there is no coating involved in plasma-etched implants, there is no risk of a coating being delaminated. Poulsson et al. [47] have produced nanometer level surface roughness on the surface of machined rod shaped PEEK implants using low-pressure oxygen plasma and tested them in sheep. Although the plasma-modified machined implants had a higher surface roughness than uncoated machined and conventional PEEK implants, no significant differences were observed in the bone-implant contact of these implants after being implanted in sheep femurs and tibia after 26 weeks [47]. A recent study by Rochford et al. suggests that oxygen plasma-treated PEEK implants promote adherence of osteoblasts even in the presence of* Staphylococcus epidermidis* [51] but the cellular interaction of these surfaces in the presence of periodontal pathogens is still unknown. It has been observed that the surface properties of plasma-treated PEEK diminish over time [41]. However, it has also been observed and reported that treating PEEK with a pulsed Nd:YAG laser before plasma treating can prolong the effects of plasma [52].

### 3.3. Electron Beam Deposition

Electron beam deposition is a process used to decompose and deposit nonvolatile fragments on a substrate [53]. A thin titanium coating deposited on PEEK using electron beam deposition has been shown to increase the wettability and promote cellular adhesion [54]. When a titanium coating on PEEK produced by electron beam deposition is anodized, it is converted into a uniformly thick (2 *μ*m), crack-free, and highly nanoporous layer of titanium oxide (nTiO_2_) which can be used to carry BMP-2 [55]. Many published* in vitro* and* in vivo* studies show that BMP-2 is a growth factor which plays a major role in differentiation of stem cells to osteoblasts [56, 57]. Given this, an immobilized growth factor on the surface of the implant could increase the rate of osseointegration around it.

### 3.4. Plasma Immersion Ion Implantation

A substrate can be coated by a thin film of diverse particles placing the substrate in a plasma of the particles, repeatedly pulsed with high negative voltages which causes the plasma ions to be accelerated and then implanted onto the surface of the substrate [58, 59]. This process is known as plasma immersion ion implantation (PIII). PEEK can be coated by nano-TiO_2_ particles using plasma immersion ion implantation [60]. A study by Lu et al. shows that PIII-coated PEEK implants could exhibit partial antimicrobial activity against* Staphylococcus aureus* and* Escherichia coli* [60]. However, it is not known if these types of surfaces could exhibit similar activity against pathogens more common in the periodontium. Furthermore, these implants have not been tested* in vivo*. Diamond-like carbon coated on PEEK has also been shown to exhibit increased bioactivity* in vitro* but the* in vivo* effects of the surface modification are yet to be evaluated [61].

## 4. Summary and Conclusion

There are many ways in which PEEK can be modified at a nanometer level to overcome its limited bioactivity. Nanoparticles such as TiO_2_, HAF, and HAp can be combined with PEEK through the process of melt-blending to produce bioactive nanocomposites. Moreover, these composites exhibit significantly superior tensile properties when compared to pure PEEK. Additionally, HAF has antibacterial properties which could prevent peri-implantitis and early implant failures. Spin-coating, gas plasma etching, electron beam deposition, and plasma-ion immersion can be used to modify or coat the surface of PEEK implants at a nanometer level. Nanocoatings of materials such as HAp and TiO_2_ produced by spin-coating and PIII can impart bioactive properties to the surface. Also, an anodized electron beam-coated TiO_2_ nanolayer on PEEK can carry immobilized BMP-2 growth factor which can further enhance cellular activity. However, many of the aforementioned studies have been limited to* in vitro* testing. Using PEEK implants, which have not undergone extensive animal and human testing, yet carries a risk of failing early. Hence, more* in vivo* studies are required before nanomodified PEEK implants can be used broadly in the clinical setting.

## Figures and Tables

**Table 1:** Bioactive PEEK nanocomposites and some biomechanical properties.

Material	Particle size	Modulus (GPa)	Tensile strength (MPa)	Contact angle (°)	Animal studies	Reference
PEEK-nTiO_2_	Not stated	3.8	93	n.d.	Yes	[30]
PEEK-nHAF	Length = 85 ± 10 nm, width: 22 ± 4 nm	12.1 ± 0.4	137.6 ± 9.1	71.5	Yes	[29]
CFR-PEEK-nHAp	<200 nm	n.d.	n.d.	75 (without plasma), 10 (with plasma treatment)	No	[62]

**Table 2:** Various surface modifications for PEEK and some reported properties.

Modification	Surface roughness/pore size	Contact angle (°)	Animal studies	References
Spin-coating				
nHAp	*S*_*a*_ = 0.686 ± 0.14 *µ*m–0.93 ± 0.25 *µ*m	53 ± 4.4	Yes	[42–44]
Gas plasma etching				
O_2_/Ar	RMS = 9–19 nm	5–40	No	[46]
NH_3_	RMS = 3–7 nm	45–90	No	[46]
O_2_	*R*_*a*_ = 75.33 ± 10.66 nm	52	Yes	[47]
E-beam TiO_2_				
Conventional	n.d.	54	No	[54]
Anodized	Pore size: 70 nm	≈0	Yes	[55]
PIII				
TiO_2_	Pore size: 150–200 nm	n.d	No	[45]
Diamond-like carbon	RMS = 5.42 nm	≈55	No	[61]

## References

[1] Awad M. A., Rashid F., Feine J. S. (2014). The effect of mandibular 2-implant overdentures on oral health-related quality of life: an international multicentre study. *Clinical Oral Implants Research*.

[2] Turkyilmaz I., Company A. M., McGlumphy E. A. (2010). Should edentulous patients be constrained to removable complete dentures? The use of dental implants to improve the quality of life for edentulous patients. *Gerodontology*.

[3] Albrektsson T., Brånemark P.-I., Hansson H.-A., Lindström J. (1981). Osseointegrated titanium implants: requirements for ensuring a long-lasting, direct bone-to-implant anchorage in man. *Acta Orthopaedica*.

[4] Brånemark P.-I. (1977). Osseointegrated implants in the treatment of the edentulous jaw. Experience from a 10-year period. *Scandinavian Journal of Plastic and Reconstructive Surgery*.

[5] Guo C. Y., Tang A. T. H., Matinlinna J. P. (2012). Insights into surface treatment methods of titanium dental implants. *Journal of Adhesion Science and Technology*.

[6] Liu D., Matinlinna J. P., Pow E. H. N. (2012). Insights into porcelain to zirconia bonding. *Journal of Adhesion Science and Technology*.

[7] Zhang M., Matinlinna J. P. (2012). E-glass fiber reinforced composites in dental applications. *Silicon*.

[8] Mallineni S. K., Nuvvula S., Matinlinna J. P., Yiu C. K., King N. M. (2013). Biocompatibility of various dental materials in contemporary dentistry: a narrative insight. *Journal of Investigative and Clinical Dentistry*.

[9] Esposito M., Hirsch J.-M., Lekholm U., Thomsen P. (1998). Biological factors contributing to failures of osseointegrated oral implants. (I). Success criteria and epidemiology. *European Journal of Oral Sciences*.

[10] Rupp F., Scheideler L., Olshanska N., de Wild M., Wieland M., Geis-Gerstorfer J. (2006). Enhancing surface free energy and hydrophilicity through chemical modification of microstructured titanium implant surfaces. *Journal of Biomedical Materials Research Part A*.

[11] Le Guéhennec L., Soueidan A., Layrolle P., Amouriq Y. (2007). Surface treatments of titanium dental implants for rapid osseointegration. *Dental Materials*.

[12] Choi A. H., Ben-Nissan B., Matinlinna J. P., Conway R. C. (2013). Current perspectives: calcium phosphate nanocoatings and nanocomposite coatings in dentistry. *Journal of Dental Research*.

[13] Brånemark P.-I., Breine U., Adell R., Hansson B. O., Lindström J., Ohlsson A. (1969). Intra-osseous anchorage of dental prostheses: I. Experimental studies. *Scandinavian Journal of Plastic and Reconstructive Surgery and Hand Surgery*.

[14] Sarot J. R., Contar C. M. M., Cruz A. C. C. D., De Souza Magini R. (2010). Evaluation of the stress distribution in CFR-PEEK dental implants by the three-dimensional finite element method. *Journal of Materials Science: Materials in Medicine*.

[15] Huiskes R., Weinans H., Van Rietbergen B. (1992). The relationship between stress shielding and bone resorption around total hip stems and the effects of flexible materials. *Clinical Orthopaedics and Related Research*.

[16] Sicilia A., Cuesta S., Coma G. (2008). Titanium allergy in dental implant patients: a clinical study on 1500 consecutive patients. *Clinical Oral Implants Research*.

[17] Siddiqi A., Payne A. G. T., de Silva R. K., Duncan W. J. (2011). Titanium allergy: could it affect dental implant integration?. *Clinical Oral Implants Research*.

[18] Becker W., Becker B. E., Ricci A. (2000). A prospective multicenter clinical trial comparing one- and two-stage titanium screw-shaped fixtures with one-stage plasma-sprayed solid-screw fixtures. *Clinical implant dentistry and related research*.

[19] Schwitalla A., Müller W.-D. (2013). PEEK dental implants: a review of the literature. *Journal of Oral Implantology*.

[20] Skinner H. B. (1988). Composite technology for total hip arthroplasty. *Clinical Orthopaedics and Related Research*.

[21] Rho J. Y., Ashman R. B., Turner C. H. (1993). Young's modulus of trabecular and cortical bone material: ultrasonic and microtensile measurements. *Journal of Biomechanics*.

[22] Yildiz H., Chang F.-K., Goodman S. (1997). Composite hip prosthesis design. II. Simulation. *Journal of Biomedical Materials Research*.

[23] Olivares-Navarrete R., Gittens R. A., Schneider J. M. (2012). Osteoblasts exhibit a more differentiated phenotype and increased bone morphogenetic protein production on titanium alloy substrates than on poly-ether-ether-ketone. *Spine Journal*.

[24] Rabiei A., Sandukas S. (2013). Processing and evaluation of bioactive coatings on polymeric implants. *Journal of Biomedical Materials Research A*.

[25] Ha S.-W., Mayer J., Koch B., Wintermantel E. (1994). Plasma-sprayed hydroxylapatite coating on carbon fibre reinforced thermoplastic composite materials. *Journal of Materials Science: Materials in Medicine*.

[26] Suska F., Omar O., Emanuelsson L. (2014). Enhancement of CRF-PEEK osseointegration by plasma-sprayed hydroxyapatite: a rabbit model. *Journal of Biomaterials Applications*.

[27] Abu Bakar M. S., Cheng M. H. W., Tang S. M. (2003). Tensile properties, tension-tension fatigue and biological response of polyetheretherketone-hydroxyapatite composites for load-bearing orthopedic implants. *Biomaterials*.

[28] Wong K. L., Wong C. T., Liu W. C. (2009). Mechanical properties and in vitro response of strontium-containing hydroxyapatite/polyetheretherketone composites. *Biomaterials*.

[29] Wang L., He S., Wu X. (2014). Polyetheretherketone/nano-fluorohydroxyapatite composite with antimicrobial activity and osseointegration properties. *Biomaterials*.

[30] Wu X., Liu X., Wei J., Ma J., Deng F., Wei S. (2012). Nano-TiO_2_/PEEK bioactive composite as a bone substitute material: in vitro and in vivo studies. *International Journal of Nanomedicine*.

[31] Huang S., Chueh P. J., Lin Y.-W., Shih T.-S., Chuang S.-M. (2009). Disturbed mitotic progression and genome segregation are involved in cell transformation mediated by nano-TiO_2_ long-term exposure. *Toxicology and Applied Pharmacology*.

[32] Wang J., Liu Y., Jiao F. (2008). Time-dependent translocation and potential impairment on central nervous system by intranasally instilled TiO_2_ nanoparticles. *Toxicology*.

[33] Sugita Y., Ishizaki K., Iwasa F. (2011). Effects of pico-to-nanometer-thin TiO_2_ coating on the biological properties of microroughened titanium. *Biomaterials*.

[34] Gittens R. A., McLachlan T., Olivares-Navarrete R. (2011). The effects of combined micron-/submicron-scale surface roughness and nanoscale features on cell proliferation and differentiation. *Biomaterials*.

[35] Wang N., Li H., Lü W. (2011). Effects of TiO_2_ nanotubes with different diameters on gene expression and osseointegration of implants in minipigs. *Biomaterials*.

[36] Wiegand A., Buchalla W., Attin T. (2007). Review on fluoride-releasing restorative materials—fluoride release and uptake characteristics, antibacterial activity and influence on caries formation. *Dental Materials*.

[37] Hamilton I. R. (1990). Biochemical effects of fluoride on oral bacteria. *Journal of Dental Research*.

[38] Tahriri M., Moztarzadeh F. (2014). Preparation, characterization, and in vitro biological evaluation of PLGA/nano-fluorohydroxyapatite (FHA) microsphere-sintered scaffolds for biomedical applications. *Applied Biochemistry and Biotechnology*.

[39] Gineste L., Gineste M., Ranz X. (1999). Degradation of hydroxylapatite, fluorapatite, and fluorhydroxyapatite coatings of dental implants in dogs. *Journal of Biomedical Materials Research*.

[40] Stanić V., Dimitrijević S., Antonović D. G. (2014). Synthesis of fluorine substituted hydroxyapatite nanopowders and application of the central composite design for determination of its antimicrobial effects. *Applied Surface Science*.

[41] Canal C., Molina R., Bertran E., Erra P. (2004). Wettability, ageing and recovery process of plasma-treated polyamide 6. *Journal of Adhesion Science and Technology*.

[42] Barkarmo S., Wennerberg A., Hoffman M. (2013). Nano-hydroxyapatite-coated PEEK implants: a pilot study in rabbit bone. *Journal of Biomedical Materials Research Part A*.

[43] Barkarmo S., Andersson M., Currie F. (2014). Enhanced bone healing around nanohydroxyapatite-coated polyetheretherketone implants: an experimental study in rabbit bone. *Journal of Biomaterials Applications*.

[44] Johansson P., Jimbo R., Kjellin P., Chrcanovic B., Wennerberg A., Currie F. (2014). Biomechanical evaluation and surface characterization of a nano-modified surface on PEEK implants: a study in the rabbit tibia. *International Journal of Nanomedicine*.

[45] Wang H., Lu T., Meng F., Zhu H., Liu X. (2014). Enhanced osteoblast responses to poly ether ether ketone surface modified by water plasma immersion ion implantation. *Colloids and Surfaces B: Biointerfaces*.

[46] Waser-Althaus J., Salamon A., Waser M. (2014). Differentiation of human mesenchymal stem cells on plasma-treated polyetheretherketone. *Journal of Materials Science: Materials in Medicine*.

[47] Poulsson A. H. C., Eglin D., Zeiter S. (2014). Osseointegration of machined, injection moulded and oxygen plasma modified PEEK implants in a sheep model. *Biomaterials*.

[48] Chan C.-M., Ko T.-M., Hiraoka H. (1996). Polymer surface modification by plasmas and photons. *Surface Science Reports*.

[49] Tsougeni K., Vourdas N., Tserepi A., Gogolides E., Cardinaud C. (2009). Mechanisms of oxygen plasma nanotexturing of organic polymer surfaces: from stable super hydrophilic to super hydrophobic surfaces. *Langmuir*.

[50] Rechendorff K., Hovgaard M. B., Foss M., Zhdanov V. P., Besenbacher F. (2006). Enhancement of protein adsorption induced by surface roughness. *Langmuir*.

[51] Rochford E. T. J., Subbiahdoss G., Moriarty T. F. (2014). An in vitro investigation of bacteria-osteoblast competition on oxygen plasma-modified PEEK. *Journal of Biomedical Materials Research A*.

[52] Akkan C. K., Hammadeh M. E., May A. (2014). Surface topography and wetting modifications of PEEK for implant applications. *Lasers in Medical Science*.

[53] Randolph S. J., Fowlkes J. D., Rack P. D. (2006). Focused, nanoscale electron-beam-induced deposition and etching. *Critical Reviews in Solid State and Materials Sciences*.

[54] Han C.-M., Lee E.-J., Kim H.-E. (2010). The electron beam deposition of titanium on polyetheretherketone (PEEK) and the resulting enhanced biological properties. *Biomaterials*.

[55] Han C.-M., Jang T.-S., Kim H.-E., Koh Y.-H. (2014). Creation of nanoporous TiO2 surface onto polyetheretherketone for effective immobilization and delivery of bone morphogenetic protein. *Journal of Biomedical Materials Research—Part A*.

[56] Wildemann B., Bamdad P., Holmer C., Haas N. P., Raschke M., Schmidmaier G. (2004). Local delivery of growth factors from coated titanium plates increases osteotomy healing in rats. *Bone*.

[57] Macdonald M. L., Samuel R. E., Shah N. J., Padera R. F., Beben Y. M., Hammond P. T. (2011). Tissue integration of growth factor-eluting layer-by-layer polyelectrolyte multilayer coated implants. *Biomaterials*.

[58] Mantese J. V., Brown I. G., Cheung N. W., Collins G. A. (1996). Plasma-immersion ion implantation. *MRS Bulletin*.

[59] Lieberman M. A. (1989). Model of plasma immersion ion implantation. *Journal of Applied Physics*.

[60] Lu T., Liu X., Qian S. (2014). Multilevel surface engineering of nanostructured TiO_2_ on carbon-fiber-reinforced polyetheretherketone. *Biomaterials*.

[61] Wang H., Xu M., Zhang W. (2010). Mechanical and biological characteristics of diamond-like carbon coated poly aryl-ether-ether-ketone. *Biomaterials*.

[62] Xu A., Liu X., Gao X., Deng F., Deng Y., Wei S. (2015). Enhancement of osteogenesis on micro/nano-topographical carbon fiber-reinforced polyetheretherketone-nanohydroxyapatite biocomposite. *Materials Science and Engineering C*.

